# Characterization of the TnsD-*attTn7 *complex that promotes site-specific insertion of *Tn7*

**DOI:** 10.1186/1759-8753-1-18

**Published:** 2010-07-23

**Authors:** Rupak Mitra, Gregory J McKenzie, Liang Yi, Cherline A Lee, Nancy L Craig

**Affiliations:** 1 Howard Hughes Medical Institute, Department of Molecular Biology and Genetics, Johns Hopkins University School of Medicine, Baltimore MD 21205, USA; 2 Current Address: Verenium Corporation. 4955 Directors Place, San Diego, CA 92121, USA; 3 Current Address: Laboratory of Host Defense, NIAID/NIH, Bethesda, MD 20892, USA; 4 Current Address: Mayo Clinic, 417 Guggenheim Bldg, 200 First St. SW, Rochester, MN 55905, USA

## Abstract

The bacterial transposon *Tn7 *is distinguished by its ability to recognize a specific site called *attTn7*, and insert just downstream of the highly conserved chromosomal *glmS *gene. TnsD is one of four transposon-encoded polypeptides (TnsABC+D) required for site-specific insertion of Tn*7 *into *attTn7*, and is the target site-selector that binds to a highly conserved sequence in the end of the *glmS *protein coding region. In this study, we identified important nucleotides within this region that are crucial for TnsD-*attTn7 *interaction. We also probed the regions of TnsD that interact with *attTn7 *and found that there are important DNA-binding determinants throughout the entire length of the protein, including an amino-terminal CCCH zinc-finger motif. A key role of TnsD is to recruit the non-sequence specific DNA-binding protein TnsC to *attTn7*; TnsC also interacts with and controls both the TnsA and TnsB subunits of the Tn7 transposase. TnsC stimulates the binding of TnsD to *attTn7 in vivo*, and TnsCD and TnsD can also interact in the absence of DNA and localize their interaction domains to the N-terminal region of each protein.

## Background

Tn*7 *is a very distinctive bacterial transposon that encodes five transposition proteins: Tns A, B, C, D and E [1]. Strikingly, whereas most transposons insert relatively randomly into many different sites, Tn*7 *transposition is quite specific. TnsD and TnsE are alternative target site-selectors that direct Tn*7 *transposition into either of two different target DNAs [2]: a very specific chromosomal attachment site or DNAs undergoing DNA replication [3].

When TnsD is the target selector, Tn*7 *inserts at high frequency into a specific chromosomal site called an attachment site, *attTn7 *[4]. Insertion occurs directly downstream of the essential *glmS *gene, ensuring that it does not disrupt the *glmS *open reading frame and *glmS *expression is preserved [5]. Thus, Tn*7 *can access this highly conserved 'safe haven' insertion site with no obvious fitness costs to the host. Tn*7 *inserts into *attTn7 *because TnsD specifically recognizes highly conserved sequences within the protein coding region of *glmS *[4], and recruits the rest of the transposition machinery to this site.

The TnsD binding site in *Escherichia coli glmS *occupies the last 36 bp of the *glmS *ORF [6]. TnsD also binds the human *glmS *homologs *gfpt-1 *and *gfpt-2 *[7]. GlmS (L-glucosamine--fructose-6-phosphate aminotransferase) is highly conserved and found in a wide variety of organisms from bacteria to humans [7]. The TnsD binding region of *glmS *encodes the active site region of GlmS, and this amino acid sequence is nearly completely (100% conserved) in all organisms [8]. Indeed, most of the DNA sequence divergence results from variation at the wobble position of each codon (see below). Intriguingly, no particular DNA sequence other than the TnsD binding site is apparently required for *attTn7 *function, even though the actual point of Tn7 insertion is about 25 bp downstream of the TnsD binding site. Changing this region in *E. coli attTn7 *[9] does not change *Tn7 *insertion frequency, and the sequences at the point of Tn7 insertion in *attTn7::Tn7 *sites in other bacteria are also distinct [10]. Furthermore, the human *glmS *homologs *gfpt-1 *and *gfpt-2 *are efficient targets for *Tn7 *insertion despite their different sequences downstream of the GlmS ORF [7]. Thus, all the sequence information necessary for *Tn7 *insertion in *attTn7 *is apparently conferred by TnsD binding to the end of *glmS*.

TnsD is a unique, sequence-specific, DNA-binding protein. It has no homologs outside of Tn*7*-type transposons, about 130 of which are now reported in Genbank. When TnsD binds to *attTn7*, another Tns protein, TnsC, is recruited [6], forming a TnsCD-*attTn7 *complex. DNA footprinting reveals that the TnsCD complex on *attTn7 *extends from the TnsD binding site to the point of Tn*7 *insertion that lies about 20 bp downstream of the stop codon of *glmS *in an intergenic region. Understanding how TnsD works is key to a detailed understanding of how the unique transposon *Tn7 *functions.

In this paper, we present results that provide insight into TnsD interactions with its *attTn7 *DNA-binding site and how TnsD functions to recruit its partner *attTn7 *binding protein TnsC. We determined the nucleotides that are important for TnsD binding in the *glmS *gene using both *in vitro *and *in vivo *assays. Our studies also revealed that in addition to binding to DNA, TnsD interacts with TnsC independently from interactions with *attTn7*. We also identified key amino acids in TnsD for DNA binding and important regions in TnsD for protein-protein interactions with TnsC. Finally, we characterized dominant-negative mutants of TnsD, which suggest that important interaction domains are distributed throughout the protein. The data show TnsD as a highly complex DNA-binding protein that regulates TnsC activity to activate Tn*7 *transposition.

## Results

### Defining important base pairs in the TnsD binding site

**Figure 1:**
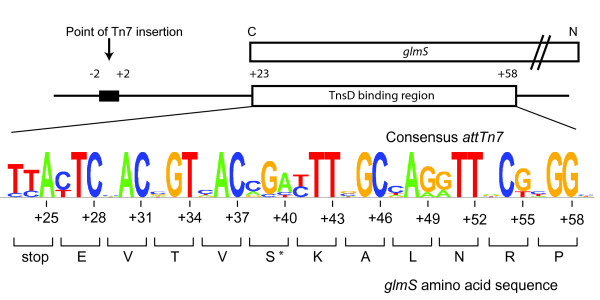
**The organization of *attTn7***. A schematic representation of the *attTn7 *at the C-terminus of the *glmS *gene with the TnsD binding site is shown. The sequence of the *E. coli *GlmS protein (asterisk indicates the only amino acid within this 11 amino acid region that is not 100% conserved within all 25 *glmS *sequences examined), and the consensus *attTn7 *sequence were derived as described in the text (note that the least conserved nucleotides correspond to the third position of each codon).

*Tn7 *insertion occurs by the attack of the 3'OH ends of *Tn7 *on staggered positions on the top and bottom strands in *attTn7*, and DNA repair of the resulting gaps, which results in 5 bp *attTn7 *duplications flanking the newly inserted *Tn7*. Throughout this paper, the middle base pair of this duplication sequence is designated '0', sequences that lie to the right towards *glmS *as '+', and those to the left as '-'; thus the *Tn7 *target site duplication is *attTn7 *-2 to +2. The minimal *E. coli *36 bp TnsD-binding site that has been defined by footprinting studies extends from *attTn7 *+23 to +58, and can promote maximum insertion activity *in vivo *and *in vitro *(Figure 1) [6]. TnsD also binds to the human *glmS *homologs *gfpt-1 *and *gfpt-2 *[7], the *Drosophila *homologs *gfat-1 *and *gfat-2*, and the zebrafish *gfpt-1 *(see Additional file 1).

The 36 bp TnsD-binding site lies within the 3' end of *glmS*, which encodes the C-terminus of the protein. This region includes the GlmS active site and is highly conserved at the protein level. We aligned this 36 bp sequence from 25 bacterial *glmS *genes that have a downstream *Tn7 *family transposon (GenBank) and the homologous *Drosophila*, zebrafish and human genes using the Sequence Logo algorithm [11] to generate a consensus for the attachment site (Figure 1). Not surprisingly for a region that encodes a highly conserved protein, the first two positions at each codon are highly conserved, and variation is seen only at the third 'wobble' position.

**Figure 2:**
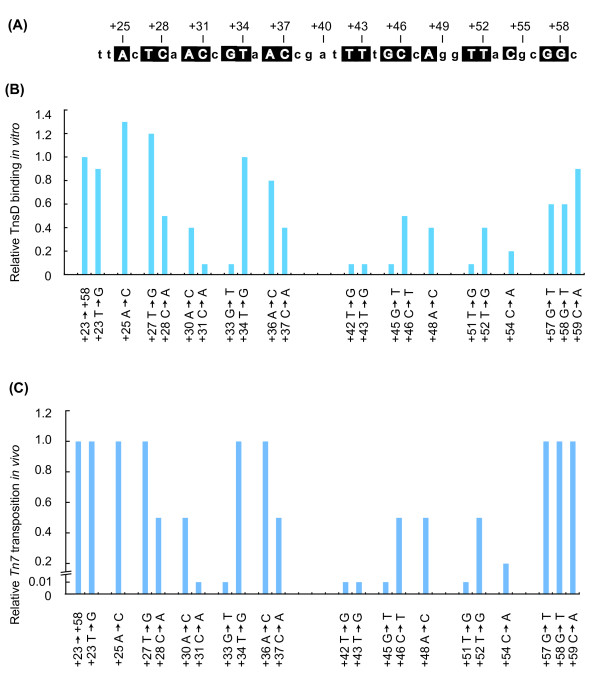
***attTn7 *target activity**. **(A) **The *E. coli attTn7 *sequence from +23 to +59 is shown; nucleotides that are highly conserved and subsequently analyzed by mutation are boxed. **(B) **Graphical representation of the relative target DNA binding activity of TnsD on the mutant *attTn7 *site *in vitro *is shown. Double stranded oligonucleotides with indicated mutations listed were used in gel-shift assays with TnsD. **(C) **Graphical representation of the relative *in vitro *transposition activity on the mutant *attTn7 *site *in vitro *is shown. The activity of mutant *attTn7 *containing plasmid as a target for *attTn7 *transposition was measured using the lambda hop assay. The activity of wt *attTn7 *is 2 × 10^-4 ^and the activity of mutants were normalized to this value.

Reasoning that TnsD would contact with the most highly conserved bases within this region, we mutated each of the most conserved bases in the *E. coli attTn7 *sequence (+23 → +58) (Figure 1, Figure 2A) to the opposite type (that is, pyrimidines to purines (e.g., cytosine to adenine) and purines to pyrimidines (e.g., guanine to thymine)). We performed gel-shift assays using purified TnsD and radiolabeled attachment-site oligonucleotides, and compared the relative amount of TnsD-*attTn7 *complex formation of the mutants with that of the wild-type attachment site (Figure 2A). Mutations at positions *attTn7 *+31, +33, +42, +43, +45, +51 and +54 all had strong negative effects; that is, they resulted in a reduction in binding of at least five-fold, indicating that those positions are very important for TnsD binding to *attTn7*. More modest (about two-fold) reductions in *attTn7 *binding activity resulted from mutation at *attTn7 *+30, +37, +48, +51 and +52.

We also examined the binding of TnsD to wild-type and representative mutant *attTn7 *sites *in vivo *(Table 1). We used the P22 challenge phage assay for DNA binding in *Salmonella*, which has been used to identify and characterize DNA-binding sites and protein-DNA interactions in a wide variety of bacterial and eukaryotic DNA-binding proteins [12]. Briefly, the anti-repressor of lysogeny (*ant*) gene of phage P22 controls the choice between P22 lysis and lysogeny upon infection. If *ant *is expressed, the phage grows lytically, whereas if expression of *ant *is blocked, lysogeny can be established. The expression of *ant *can be blocked by engineering the binding site for a heterologous DNA-binding protein (here *attTn7*), upstream of *ant *and providing the cognate binding protein (here TnsD), in *trans*. If binding of the tested protein to the introduced site occurs, expression of *ant *is blocked and lysogeny ensues, which can be scored by the number of kanamycin-resistant colonies, as the P22 genome imparts kanamycin resistance to the cell. Thus, P22 lysogeny can be a measure of protein binding; lysogeny reflects binding of TnsD, whereas if TnsD does not bind, *ant *is expressed and the phage grows lytically.

**Table 1:** TnsD binding to *attTn7 in vivo *

*attTn7* position	**Lysogeny frequency**^**a**^	
	
	TnsD induced	TnsD uninduced
+23 → +58(wt)	7.4 × 10^-5^	5.4 × 10^-7^

+31 C→A	5.2 × 10^-9^	-

+42 T→G	3.6 × 10^-8^	-

+43 T→G	1.6 × 10^-8^	-

+51 T→G	5.2 × 10^-9^	-

^a^Challenge assay phages carrying the indicated mutations in *attTn7 *were infected into cells expressing TnsD and the frequency of lysogeny measured; values are the average of three independent infections performed in parallel and relative to the wild type.

We know from previous work that *Tn7 *transposition functions well in *Salmonella *[13]. For the challenge phage experiment, we constructed a P22 phage with the *E. coli attTn7 *attachment site (+23 → +58) upstream of *ant*, thus putting the choice of lysogenic vs lytic growth under the control of TnsD binding to *attTn7*: if TnsD binds, lysogeny should occur. We infected a *Salmonella enterica *Typhimurium strain containing a plasmid-borne isopropyl β-D-1-thiogalactopyranoside (IPTG)-inducible *tnsD *gene with the challenge phage carrying the *E. coli *attachment site upstream of *ant*. The frequency of lysogenization of the *attTn7 *phage increased more than 100-fold in the presence of TnsD (Table 1). Thus the challenge phage assay can be successfully used to evaluate TnsD binding to *attTn7 in vivo*. When assayed, the frequency of lysogeny of a phage containing a wild-type attachment site (+23 → +58) was several orders of magnitude higher than the frequency of lysogeny of phages containing any of four mutant attachment sites that bind TnsD poorly *in vitro *(Table 1). These assays show that TnsD also binds poorly to these mutant sites *in vivo*, supporting the view that the identity of these positions is important for the site-specific binding activity of TnsD.

### *attTn7 *mutations that block TnsD binding also block *Tn7 *transposition *in vivo*

As described above, mutations in *attTn7 *can block TnsD binding both *in vitro *and *in vivo*. We also determined the effect of *attTn7 *mutations on *Tn7 *insertion into *attTn7 in vivo *in *E. coli*. In these assays, *Tn7 *transposition into a target plasmid containing *attTn7 *was evaluated using a 'λ-hop' assay [14], in which cells containing a plasmid(s) expressing TnsABC+D were infected with a replication- and integration-defective lambda phage derivative carrying a mini*Tn7 *kanamycin resistance cassette (miniTn*7*-Kan^R^), and the frequency of mini*Tn7 *insertion into the *attTn7 *plasmid was measured as the fraction of infected cells that became kanamycin-resistant.

We found that mutations that significantly (> 10-fold) decreased TnsD binding to *attTn7 *also decreased *Tn7 *transposition *in vivo *by at least 100-fold (Figure 2C). Thus, *attTn7 *+31, +33, +42, +43, +45 and +51 are key positions for TnsD-*attTn7 *interaction. *attTn7 *sequence changes that had more modest effects on TnsD binding, (reductions of two- to five-fold), also reduced transposition by two- to five-fold. Thus, *attTn7 *+28, +30, +37, +46, +48 and +54 also make contributions to TnsD-*attTn7 *interaction.

### Crosslinking of *attTn7 *to TnsD

**Figure 3:**
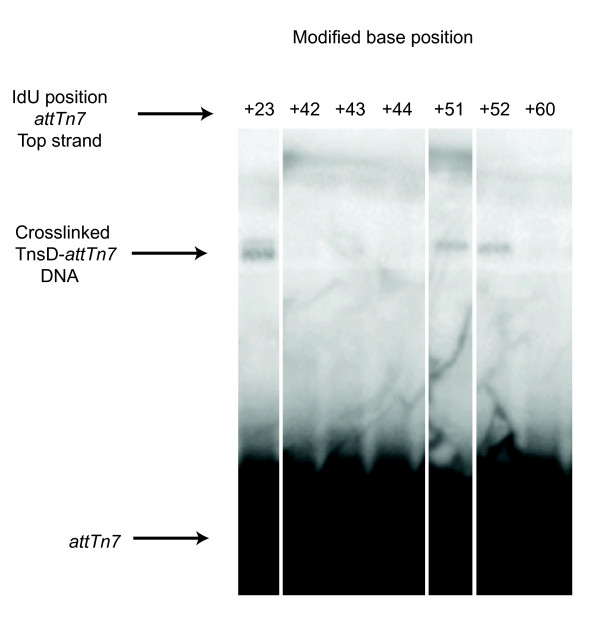
**Crosslinking between TnsD and *attTn7***. Thymine was replaced with iodouracil (IdU) and photocrosslinked to TnsD, followed by SDS-PAGE. Free and bound DNA are indicated for different positions along the attachment site.

Crosslinking studies with radiolabeled *attTn7 *DNA were also used to further probe TnsD-*attTn7 *interaction. Each 'T' on both strands of the attachment site was individually replaced with iodouracil, then the *attTn7 *derivatives were tested for binding to TnsD by bandshift assay and crosslinking was performed in the presence of UV light. None of these iodouracil substitutions had a detectable effect on TnsD binding *in vitro *(data not shown). Of all of the positions tested, only IdUs at positions at *attTn7 +*23, +51 and +52 were in sufficiently close proximity to TnsD to produce covalent protein-DNA bonds in this assay (Figure 3); in all these cases, the crosslinkable base was on the top strand of *attTn7*. As described above, mutation alterations of the attachment site at both *attTn7 *+51 and +52 had a negative effect on TnsD binding to *attTn7*. These results suggest that there are important base-specific contacts between *attTn7 *and TnsD at these positions. By contrast, mutation of the crosslinkable position *attTn7 *+23 had no effect on TnsD binding or target activity *in vivo*. It should be noted that in such an assay, crosslinking requires several different amino acids to be juxtaposed, thus a failure to observe crosslinking does not mean that TnsD is not immediately adjacent to DNA at these positions, but rather that no crosslinkable amino acids are nearby.

### TnsD contains a zinc-finger motif essential for DNA binding

**Figure 4:**
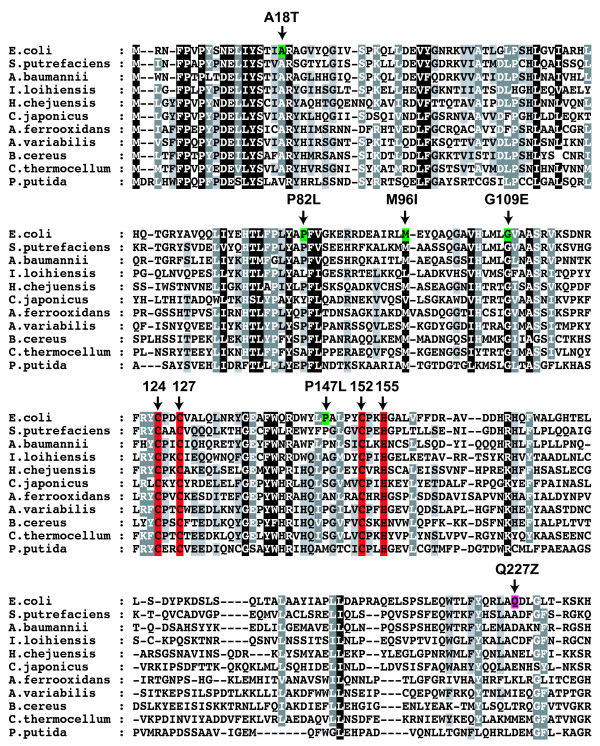
**Multiple sequence alignment of TnsD proteins**. An alignment of 11 different TnsD proteins (produced using the software T-Coffee; http://www.ebi.ac.uk/t-coffee) showing the highly conserved CCCH zinc finger motif and the isolated dominant-negative mutants with reference to *E. coli *TnsD protein, amino acid positions 1 to 213.

**Figure 5:**
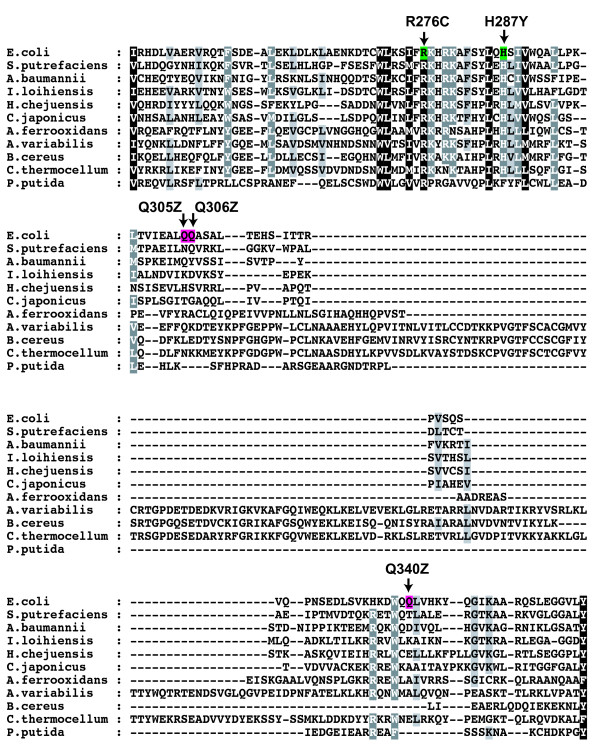
**Multiple sequence alignment of TnsD proteins**. An alignment of 11 different TnsD proteins (produced using the software T-Coffee; http://www.ebi.ac.uk/t-coffee) showing the isolated dominant-negative mutants with reference to *E. coli *TnsD protein, amino acid positions 214 to 323.

**Figure 6:**
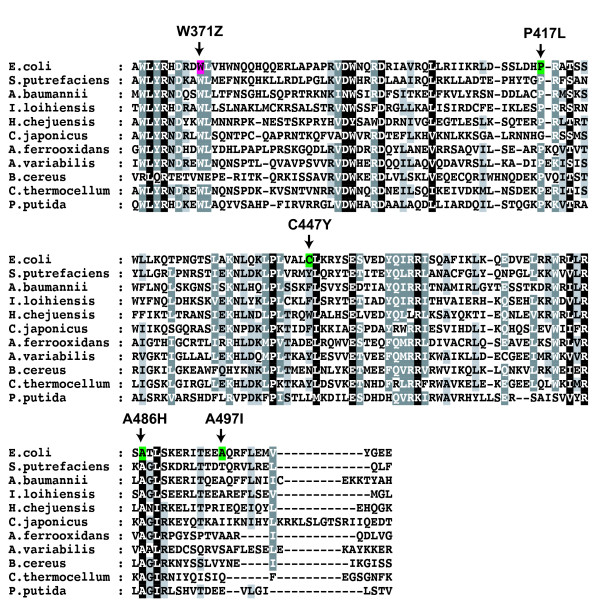
**Multiple sequence alignment of TnsD proteins**. An alignment of 11 different TnsD proteins (produced using the software T-Coffee; http://www.ebi.ac.uk/t-coffee) showing the isolated dominant-negative mutants with reference to *E. coli *TnsD protein, amino acid positions 324 to 507.

Before the modern era of extensive bacterial genome sequencing, *Tn7*, which was identified in *E. coli *[15,16], was unique. Now there are many examples in GenBank of *Tn7 *relatives in a variety of different bacteria that contain obvious TnsD homologs (Figure 4, Figure 5, Figure 6). The most notable feature of this alignment is a highly conserved N-terminal region of about 170 amino acids that contains a CCCH motif characteristic of zinc-finger proteins [17]. In *E. coli Tn7 *TnsD, the motif consists of Cys^124^, Cys^127^, Cys^152 ^and His^155 ^(Figure 4).

**Figure 7:**
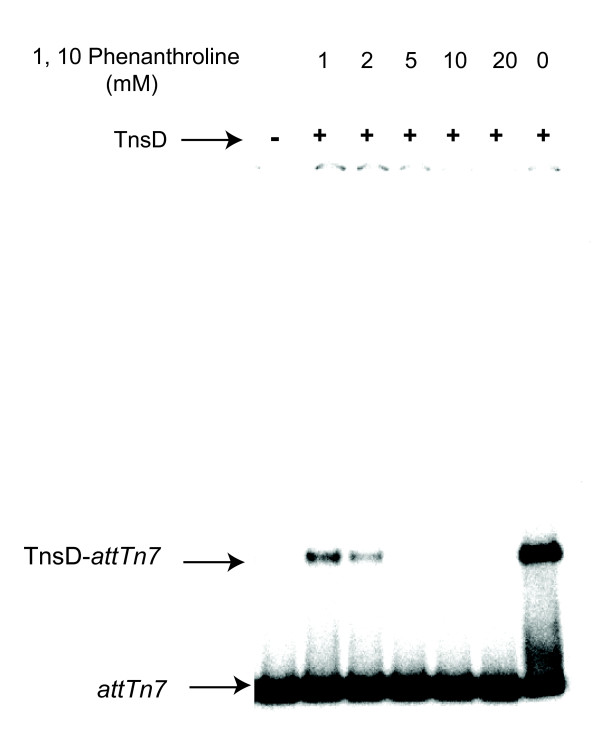
**Chelation of zinc using 1,10-phenanthroline eliminates TnsD binding to *attTn7 *DNA**.

To determine directly whether TnsD binding to *attTn7 *is dependent on zinc, gel-shift assays were performed using wild-type TnsD and *attTn7 *in the presence of increasing amounts of the zinc chelator 1,10-phenanthroline. Complex formation was severely diminished by 2 mM 1,10-phenanthroline, indicating that TnsD DNA binding is zinc-dependent (Figure 7). We also individually mutated Cys124, Cys127, Cys152 and His155 to serine, and analyzed the ability of these mutants to bind *attTn7 in vitro *and to promote transposition *in vivo *(Table 2). When purified, none of the mutant TnsD proteins were able to bind *attTn7 *(Table 2). We again used the 'λ-hop' assay to assay transposition of a mini*Tn7*-Kan^R ^[14] from the infecting phage into an *attTn7 *plasmid, by measuring the fraction of infected cells that become kanamycin-resistant. The frequency of transposition frequency promoted by the TnsD zinc-finger mutants was reduced 1000-fold from that of wild-type cells. Sequencing of junctions of independent insertions from the *in vivo *transposition assay showed that *Tn7 *insertions were present in *attTn7 *in a sequence- and orientation-specific manner, with the 5-bp target duplication characteristic of *Tn7 *transposition, as occurs with wild-type TnsD (data not shown). Thus, the insertions recovered, although occurring at very low frequency, were probably TnsD-mediated transposition events. As described in more detail below, we were unable to observe DNA binding *in vitro *with various N-terminal fragments of TnsD, thus could not identify a discrete zinc finger motif-containing DNA-binding domain.

**Table 2:** Targeted mutations to the conserved CCCH residues in TnsD eliminates *Tn7 *transposition and TnsD-*attTn7 *binding.

Protein	Relative *attTn7 *binding *in vitro*	Relative transposition frequency *in vivo*
TnsD wt	1.0	1.0^a^

TnsD C124S	0.05	< 0.001

TnsD C127S	0.05	< 0.001

TnsD C152S	0.05	< 0.001

TnsD C155S	0.05	< 0.001

^a^The transposition frequency in the presence of TnsD wt is 7.8 × 10-4.

### TnsD has important DNA-binding determinants throughout the protein

In an attempt to identify directly the domains of TnsD involved in DNA binding, we generated a number of TnsD deletion derivatives, and assessed DNA binding of these mutants *in vivo *and *in vitro*. All tested C-terminal deletion derivatives, (TnsD1 to 350, 1 to 399, 1 to 480, 1 to 490 and 1 to 498) were unable to bind DNA as evaluated by *in vitro *band-shift assays and by *in vivo *challenge phage assays (data not shown). However, these truncated proteins did retain the ability to interact with TnsC, indicating that they were not simply unfolded (we discuss interaction of TnsD with TnsC below). These observations suggest that in addition to the zinc finger, TnsD contains DNA-binding determinants spread across the entire length of the protein and that several of these are required for *attTn7 *binding. We were unable to detect interaction of N-terminal TnsD truncations with TnsC or to purify these proteins (data not shown).

### Isolation of TnsD dominant-negative mutations

In another approach to identify the functional regions of TnsD, we conducted a screen for mutations in TnsD that have dominant-negative effects on TnsD-dependent transposition. Expected classes of TnsD dominant-negative mutants include those able to bind *attTn7 *but unable to interact with TnsC, and also mutants unable to bind *attTn7 *but able to interact with TnsC. We used a promoter capture (papillation) assay as described by Stellwagen and Craig[18], in which a *miniTn7 *containing a promoterless *lac *gene is mobilized from a plasmid donor to chromosomal pseudo-*attTn7 *sites; *attTn7 *itself was blocked by another *Tn7 *element. TnsABC+D were expressed from a pACYC plasmid. Productive hops produce Lac^+ ^cells that form papillae on Lac^- ^colonies on MacConkey lactose plates. To look for dominant-negatives, we used hydroxylamine to mutate a pUC-based *TnsD *gene, transformed it into the assay strain containing wild-type TnsABC+D, and looked for transformants on MacConkey lactose plates within decreased numbers of papillae. After hydroxylamine mutagenesis of *TnsD *and screening of 15,000 transformants, we isolated 16 TnsD mutants, 11 of which were missense mutants and five of which were nonsense mutants yielding truncated TnsD; only two mutants (TnsD^P147L ^and TnsD^R276C ^) were isolated twice (Figure 4, Figure 5, Figure 6).

We also quantitatively evaluated the effect of the dominant-negative TnsD mutants on transposition. Using the λ hop assay, we assayed mini*Tn7*-Kan^R ^insertion into chromosomal *attTn7 *in the presence of pACYC TnsABC+D and a pUC-based plasmid containing the TnsD mutants (Table 3). In this assay, all of the dominant-negative mutants identified by the papillation screen with the exception of TnsD C447Y inhibited TnsABC+D transposition at least 10-fold, confirming that they were indeed dominant-negatives. Although the dominant-negative effect of TnsD C447Y was more modest, this may reflect the fact that the insertion targets are different in these assays: the papillation assay measures insertion into pseudo-*attTn7 *sites, whereas the λ-hop assay measures insertion into chromosomal *attTn7*.

**Table 3:** Dominant-negative mutants of TnsD.

**From dominant-negative TnsD screen**^**a**^	Relative transposition in presence of		**Relative *attTn7 *binding *in vivo***^**d**^	Relative *attTn7 *binding *in vitro*
		
TnsD proteins	**TnsABC**^**b**^	**TnsABC**^**c**^		
No TnsD	1.00	0	< 0.00001	-

TnsD WT	1.00	1.13	1.0	1.0

TnsD A18T	0.05	0.01	-	-

TnsD P82L	0.05	0.01	-	-

TnsD M96I	0.10	0.30	0.005	0.3

TnsD G109E	0.07	< 0.001	0.0006	0.05

TnsD P147L	0.05	< 0.001	0.0016	0.07

TnsD R276C	0.04	< 0.001	-	-

TnsD H287Y	0.06	< 0.001	-	-

TnsD P417L	0.08	0.04	0.0014	0.07

TnsD C447Y	0.10	0.40	0.012	0.7

TnsD A486H	0.06	0.04	-	0.1

TnsD A497I	0.06	0.04	0.0005	0.05

Truncations				

TnsD Q227Z	0.05	< 0.001	-	-

TnsD Q305Z	0.03	< 0.001	-	-

TnsD Q306Z	0.03	< 0.001	-	-

TnsD Q340Z	0.04	< 0.001	0.0004	< 0.05

TnsD W371Z	0.08	< 0.001	-	< 0.05

^a^These substitutions and truncations were isolated as dominant negatives and then analysed for transposition *in vivo *and DNA binding *in vivo *and *in vitro*.

^b^Tn7 transposition frequency *in vivo *was measured using the lambda hop assay and carried out in presence of TnsABCD + TnsD (dominant-negative mutants).

^c^Tn7 transposition frequency *in vivo *was measured using the lambda hop assay and was carried out in presence of TnsABC + TnsD (dominant-negative mutants).

^d^The binding of mutant TnsDs to *attTn7 in vivo *was measured using the challenge phage assay.

We also examined the ability of the dominant-negative TnsD mutants to promote transposition in the presence of TnsABC alone to determine if they possess residual TnsD activity. All had substantial defects in TnsD activity, with reductions ranging from at least 2.5-fold to 100-fold compared with wild-type cells.

Using the challenge phage assay, we also evaluated the ability of most of the dominant-negative TnsD missense mutants to bind to *attTn7 in vivo*, and found that all were significantly reduced in their ability to bind *attTn7 *(Table 3). Using purified TnsD, we also found that all the missense mutants except C447Y had significantly impaired *attTn7 *binding *in vitro *(Table 3).

### TnsC increases the binding of TnsD to *attTn7*

TnsC and TnsD form a novel complex with *attTn7 in vitro*, and the presence of TnsC increases the amount of protein-DNA complex formed [6]. We also used the challenge phage assay to examine the TnsC-TnsD-*attTn7 *interaction *in vivo *(Table 4). TnsC does not promote the formation of lysogens on its own, as expected given its non-specific DNA-binding activity [19]. However, when TnsC and TnsD were expressed simultaneously in the host cells, we observed a enhancement of lysogeny of nearly 100-fold relative to cells expressing only TnsD. Thus, TnsC considerably enhances TnsD binding to *attTn7 both in vivo *and *in vitro*.

**Table 4:** TnsC increases TnsD binding to *attTn7 in vivo*

Tns protein	**Lysogeny frequency**^**a**^
TnsC	< 8.0 × 10^-7b^

TnsD	2.4 × 10^-3^

TnsC + TnsD	2.4 × 10^-1^

^a^Using the *in vivo *challenge phage assay, adding TnsC to TnsD increased lysogeny, also indicating that TnsC is stabilizing TnsD on the DNA. TnsC alone did not increase lysogeny.

^b^Lowest possible measurement in this assay.

Although the binding of wild-type TnsD to *attTn7 *is stimulated by TnsC, incubation with TnsC did not 'rescue' the formation of TnsCD-*attTn7 *complexes with the dominant-negative missense mutant TnsD *in vitro *(data not shown).

### TnsC and TnsD can interact in the absence of *attTn7*

Previous studies revealed that a key determinant in the recruitment of TnsC to TnsD-*attTn7 *is a distortion introduced by TnsD into *attTn7*, which provides a binding site for TnsC [6]. We have now found, using a yeast two-hybrid assay, that TnsC and TnsD also interact directly in the absence of *attTn7 *[20,21] (Table 5). The interaction between full-length TnsC and TnsD is only slightly less than that of the robust interaction displayed by the Fos-Jun control, but equivalent to that of RB and E2F1 [22].

**Table 5:** Yeast 2 hybrid assay shows that TnsC and TnsD can interact *in vivo*.

Protein fragment		β-galactosidase activity
Fos	Jun	5.0

E2F1	RB	1.0

Empty vector Gal4-AD	Empty vector Gal4-DB	0.075

TnsC (1 to 555) wt	TnsD (1 to 507) wt	1.0

TnsC (1 to 555) wt	TnsD (1 to 436)	0.075

TnsC (1 to 555) wt	TnsD (1 to 399)	4.5

TnsC (1 to 555) wt	TnsD (1 to 375)	5.0

TnsC (1 to 555) wt	TnsD (1 to 350)	4.5

TnsC (1 to 555) wt	TnsD (1 to 309)^a^	4.5

TnsC (1 to 555) wt	TnsD (1 to 293)	0.075

TnsC (1 to 555) wt	TnsD (8 to 507)	0.075

TnsC (1 to 555) wt	TnsD (12 to 507)	0.075

TnsC (1 to 555) wt	TnsD (16 to 507)	0.075

TnsC (1 to 555) wt	TnsD (22 to 507)	0.075

TnsC (1 to 555) wt	TnsD (1 to 507) wt	1.0

TnsC (1 to 480)	TnsD (1 to 507) wt	1.0

TnsC (1 to 384)	TnsD (1 to 507) wt	1.0

TnsC (1 to 332)	TnsD (1 to 507) wt	0.20

TnsC (1 to 293)^a^	TnsD (1 to 507) wt	0.20

TnsC (1 to 280)	TnsD (1 to 507) wt	0.014

^a^Deletion analysis of TnsD and TnsC revealed that this protein-protein interaction activity is contained within TnsC 1 to 293 and TnsD 1 to 309.

As determined by analysis of a series of C-terminal deletion derivatives, the amino-terminal amino acid sequence (1 to 309) of TnsD retain full binding to TnsC in this assay and thus contains the determinants for TnsD binding to TnsC. The isolation of the TnsD truncation sequence (1 to 340; full-length TnsD is 507 aa) as a dominant negative is consistent with this hypothesis (see below). However, if as few as seven amino acids are removed from the N-terminus of TnsD, the TnsC-TnsD interaction is disrupted (data not shown). The lack of activity of TnsD sequence 1 to 436, even though both longer and shorter constructs are active, may be due to inappropriate folding or to the unmasking of an inhibitory domain.

We also performed deletion analysis of TnsC using the yeast two-hybrid system (Table 5). TnsC-TnsD interaction with TnsD is maintained with deletion of 75 or 171 amino acids from the C terminus of TnsC (full-length is 555 aa), but a deletion of 223 amino acids leads to loss of the interaction with TnsD (Table 5); thus interaction of TnsC with TnsD occurs within the 1 to 293 region of TnsC. The finding that Tn7 inserts at high-frequency in *attTn7 *using TnsC 86 to 555 (Spencer J and NLC, unpublished observation) suggests that the TnsD interaction domain lies within TnsC 1 to 293.

## Discussion

The sequence-specific binding of TnsD to the 3' end of the coding region of the *glmS *gene is sufficient to prompt the site - and orientation-specific insertion of *Tn7 *to a position about 20 bp downstream of *glmS*, that is, to produce a functional *attTn7 *site [23]. Previous work *in vitro *identified the 36 bp span of the TnsD binding site, and demonstrated that this sequence alone is sufficient to direct transposition [6]. In this study, we have further probed the interaction of TnsD with *attTn7 *and deepened our understanding of *Tn7 *site-specific insertion. Our results have revealed that determinants of the interaction of TnsD with *attTn7 *extend throughout the TnsD protein and the TnsD binding site.

**Figure 8:**
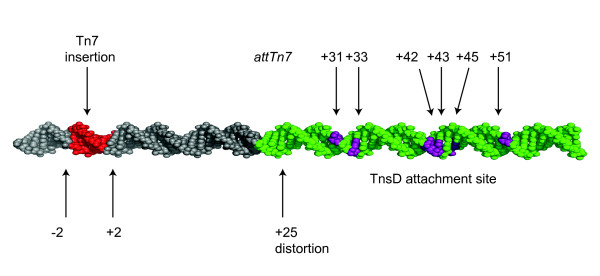
**DNA model of *attTn7***. A model of the attachment site, showing the minimum *attTn7 *site region required for TnsD binding in green, important bases (of one strand) in purple, unimportant sequence in gray, and the target duplication site in red. Phosphates are marked for clarity, although binding is probably dependent on base interactions, not sugar-backbone interactions.

The amino acid sequence of the C-terminus of virtually all GlmS proteins is identical. Comparison of the TnsD binding site in *attTn7 *sites from a variety of bacteria and several eukaryotes revealed that 19 of the 35 bp were completely conserved, and correspond to non-wobble positions for each codon at the *glmS *terminus. Analysis of TnsD binding and transposition to these mutant sites with substitutions at each of these conserved positions revealed that changes at seven (*attTn7 *+31, +33, +42, +43, +45, +51 and +54) had significant effects on TnsD binding and *Tn7 *transposition. Modeling of the attachment site as B-form DNA (Figure 8) indicates that the important nucleotides are on one face of the DNA. This is consistent with the crosslinking data presented here, in which contacts with the major groove were detected (Figure 3), and with previous DNA footprinting studies showing that TnsD makes major groove contacts [6].

The activity of *attTn7 *is strongly directional: *Tn7 *always inserts downstream of the *glmS *termination codon, mediated by activation of a TnsCD-*attTn7 *complex that promotes orientation-specific insertion of *Tn7*. Consistent with this asymmetric pattern of insertion, *attTn7 *does not contain inverted repeats for the binding of an oligomeric TnsD.

Previous studies have shown that a key step in *Tn7 *insertion into *attTn7 *is the ability of TnsD to introduce a DNA distortion(s) into *attTn7*, which we have proposed prompts the binding of TnsC, the regulator of the TnsAB transposase [6,24]. In addition to providing a higher resolution view of TnsD-*attTn7 *interaction, we also identified a previously unknown interaction protein-protein interaction between TnsC and TnsD. Determinants for this interaction are located within the N-terminal region of TnsD covering positions 1 to 309 and the N-terminal region of TnsC covering positions 1 to 293. It seems likely that the DNA-mediated and protein-protein interactions are both important to TnsC-mediated activation of transposition.

TnsC also interacts with and activates both subunits of the Tn*7 *transposase (TnsA and TnsB), which mediate breakage and joining of the ends of Tn*7 *[25]. TnsA, the transposase subunit that cleaves at the 5' ends of *Tn7*, and TnsC form a TnsA_2_C_2 _complex in solution, mediated by interactions between the N-terminal region of TnsA and the 50 carboxy-terminal amino acids of TnsC [26]. A distinct TnsACD complex has also been observed [27], which we suggested is the 'target' DNA complex, with which the TnsB transposase subunit mediates breakage and joining at the 3' ends of the transposon [28]. The other transposase subunit TnsB binds specifically to the ends of *Tn7*, and mediates breakage and joining at the 3' ends [29]. Interactions between TnsC and the C-terminus of TnsB have also been detected [30]. The apposition of the TnsACD-*attTn7 *and TnsB-*Tn7 *end complexes results in the assembly and activation of the transposase [28,31]. Much remains to learned about the mechanism by which TnsD-*attTn7 *activates TnsC and hence TnsAB.

### Functional domains of TnsD

TnsD is a multifunctional protein: it binds specifically to *attTn7 *and, as we have shown here, interacts with its partner in transpositon, TnsC. In this study, we have made considerable progress in understanding the unique TnsD family of DNA-binding proteins by defining important protein determinants for DNA binding and transposition, and key nucleotides for the TnsD-*attTn7 *interaction. Although we identified a zinc finger motif in the N-terminal region of TnsD, we were unable to use deletion analysis to identify a functional discrete DNA-binding domain(s). It is possible there are multiple DNA-binding domains throughout the protein, a model consistent with the fact that the region of TnsD interaction with DNA spans about 35 bp. However, we were able to use deletion analysis to localize the TnsC-interaction region of TnsD to its N-terminal amino acids 1 to 309, and the TnsD-interaction region of TnsC to its amino acids 1 to 293. Although our ability to isolate TnsD dominant negatives is consistent with this protein being multifunctional, our inability to further identify crucial subregions within TnsD precludes definitive conclusions of the physical basis of the dominant negatives.

As previously proposed, the ability of TnsD to direct transposition to a defined sequence makes it an attractive candidate for use in targeted delivery of DNA sequences for genetic manipulation in organisms from bacteria to human [7,13]. Further structure-function analyses of TnsD will not only facilitate a deeper understanding of *Tn7 *transposition but also the use of *Tn7 *and TnsD as tools for genomic engineering.

## Methods

### Bacterial strains, plasmids and phages

CAG456 *is E. coli *(SC122 *htpR165*) [32]. NLC51 is *E. coli *F^-^*araD139 *Δ*(argF-lac)U169 rpsL150 relA1 flbB5301 deoC1 ptsF25 rbsR valR recA56 *[33]. BD409 is a derivative of *E. coli *CW51 with *att*::promoterless *lacZY *flanked by *Tn7 *transposon end sequences sufficient for transposition: 166 bp from the left end of Tn*7*(Tn*7*L) and 90 bp from the right end of (Tn*7*R) [4,34]. Plasmid pMR1 was constructed by PCR amplifying the *tnsD *gene from pCW4 [4] and cloning it into pCYB1 digested with *Nco*I and *Sap*I (New England Biolabs, Ipswich, MA, USA.). pCW4 was used as a source of TnsABCD and pCW15 as a source of TnsABC in the *in vivo *transposition assays [4]. pCW23 was used for mutating *tnsD *[4]. Phage KK1, used as the source of Tn*7 *transposon in the λ-hop assay, is a derivative of 780 (*b*2::*hisOGD b522 cI857 Pam80 nin5*) with *hisG9424*::Tn*10 del*16 *del*17::*attTn7*(-342 to +165)::miniTn*7*kan^R ^[35].

### Affinity purification of intein-TnsD

The full length TnsD wild-type and mutant proteins were purified as intein fusions from CAG456 containing plasmid pRM1 or mutant TnsD derivative plasmids. Site-directed mutations in *tnsD *were generated by PCR (QuickChange Site Directed Mutagenesis System; Agilent Technologies) and verified by direct DNA sequencing. Representative cells were grown at 30°C to an OD_600 _= 0.5 in Luria broth supplemented with 100 mg/ml carbenicillin, IPTG was added to give 0.4 mM final concentration, and the cells were allowed to grow for an additional 4 h. All subsequent steps were performed at 4°C unless otherwise stated. The cells were separated by centrifugation, and resuspended in the buffer supplied with the kit (Buffer A; 50 mM HEPES pH 8, 500 mM NaCl, 10% v/v glycerol). The cells were then lysed by sonication and separated by centrifugation at 26,000 g for 30 min, the resulting supernatant was then filtered through a 0.45 μm syringe filter (Nalgene, Rochester, NY, USA). The filtrate was applied to pre-equilibrated chitin beads (New England Biolabs) in a 10 ml column and the beads then washed several times (5×) in Buffer A. The washed chitin beads were then treated with Buffer B (50 mM HEPES pH 8, 500 mM NaCl, 10% v/v glycerol, 10 mM MgCl_2_, 10 mM ATP) for 1 h at room temperature to remove residual GroEL protein. The beads were then washed with several volumes of buffer B at 4°C (2×), and incubated overnight with Buffer C (50 mM HEPES pH 8, 500 mM NaCl, 10% v/v glycerol, 50 mM dithiothreitol (DTT)), which promotes cleavage of TnsD from the intein tag [36,37]. Full-length TnsD was then eluted from the column using Buffer C without DTT, and peak fractions were pooled, dialyzed against another buffer (500 mM KCl, 50 mM Tris-HCl (pH 8.0), 1 mM EDTA, 2 mM DTT and 25% v/v glycerol), then stored at -80°C.

### Isolation of dominant-negative *tnsD *mutants

pCW23 (TnsD) [4] was treated with 1 M hydroxylamine hydrochloride in NaOH at 37°C for 24 h. The mutagen was dialyzed out of the DNA in Tris-EDTA buffer. The DNA was recovered by ethanol precipitation, and transformed into BD409 carrying wild-type *tnsABCD *on a compatible plasmid, pCW4 [4]. Transformants were plated onto MacConkey lactose plates containing appropriate antibiotics. The plates were incubated at 30°C for 5 days, and transformants were screened for decreased papillation (to Lac^+^). Plasmid DNA was extracted from these potential mutants and transformed into the same strain background to verify the papillation phenotype. DNA was sequenced to identify the mutations, and recloned for expression.

### λ hop assay

The transposition frequency of a miniTn*7*kan^R ^element from the integration- and replication-defective phage KK1 into the chromosomal *attTn7 *site of *E. coli *strain NLC51 and a plasmid containing the *attTn7 *site of *E. coli *strain LA3 was evaluated when Tns proteins were supplied *in trans *[35,38] The transposition frequency was the number of kanamycin-resistant colonies per plaque-forming unit.

### DNA-binding reactions

Binding reactions were performed with wild-type and mutant versions of TnsD proteins and wild-type TnsC protein as described previously [6].

### Protein-DNA UV photo-crosslinking reaction

Protein-DNA crosslinking was performed as described previously [39].^. ^Oligonucleotides were synthesized (Eurofins MWG Operon, Huntsville, AL, USA) with dT replaced by IdU. Double-stranded (ds)DNAs were then formed by gradual annealing with a complementary oligonucleotide, and then radio-labeled with 5' phosphorylation.

For crosslinking reactions, 25 pmol TnsD and 0.5 pmol *attTn7 *dsDNA were incubated in solution (25 mM HEPES, 2.5 mM TrisCl, 40 mM DTT, 0.005% BSA and 50 ng/ul herring sperm DNA, pH 7.5) for 20 min at 30°C. Photo-crosslinking was then carried out with UV irradiation at 312 nm (StrataLinker; Agilent Technologies, La Jolla, CA, USA) for 30 min. The reaction was set up so that the top of the open 1.5 ml Eppendorf tube touched the UV tube. After UV crosslinking, the reaction was analyzed by SDS-PAGE.

### Yeast two-hybrid assay

The yeast two-hybrid assay was performed according to the manufacturer's protocol (Proquest Two-Hybrid System; Life Technologies Inc., Carlsbad, CA, USA). TnsD was cloned into pDBLeu (Life Technologies Inc) and TnsC into pPC86 (Life Technologies Inc). β-galactosidase assays were performed as described previously [40].

### *In vitro *transposition reactions

I*n vitro *transposition reactions were performed essentially as described previously [19]. The donor plasmid pEMΔ (5.9 kb) contains a 1.6 kb miniTn*7*kan^R ^element. The 3.2 kb target plasmid pRM2 [35] contains a 555 bp *attTn7 *segment (-342 to +165). Reaction mixtures (100 μl final volume) contained 0.25 nM pEM donor DNA, 2.5 nM pRM2 target plasmid, 28 mM HEPES pH 8.0, 2.2 mM DTT, 4.4 mM Tris pH 7.5, 100 mg/ml tRNA, 50 mg/ml bovine serum albumin (BSA), 0.16 mM EDTA, 0.1 mM MgCl_2_, 0.1 mM CHAPS detergent, 30 mM NaCl, 21 mM KCl, 1.8% v/v glycerol, 2.0 mM ATP and 15 mM MgOAc unless otherwise indicated. Tns proteins were added as follows: 40 ng TnsA, 25 ng TnsB, 30 ng TnsC and 22 ng TnsD. Reaction mixtures containing all components except donor DNA, TnsA, TnsB and MgOAc were assembled on ice. The assembly reaction mixtures were incubated for 20 min at 30°C; donor DNA, TnsA, TnsB and MgOAc were then added, and the incubation was continued for an additional 20 min. Reactions were stopped by adjusting to 25 mM EDTA, followed by extraction with phenol:chloroform (1:1). The DNA was then precipitated with ethanol, digested with *Nde*I and separated in a 0.6% agarose gel, with electrophoresis carried out at 50 V for 16 h. The DNAs were transferred to a hybridization membrane (Gene Screen Plus; PerkinElmer, Foster City, CA, USA) and hybridized with a probe specific for miniTn*7*kan^R^. The probe was labeled by random priming with ^32^P-dCTP and the Klenow fragment of DNA polymerase I (Boehringer Mannheim Biochemicals (BMB), Indianapolis, IN, USA). All blots were analyzed using a phosphorescent imager (PhosphorImager; Molecular Dynamics, Sunnyvale, CA, USA).

### Challenge phage assays

Challenge phages were constructed as described in [12]. The wild-type *attTn7 *attachment site *attTn7 *(+23 → +58) was cloned in as oligonucleotides (5'-CCGCGTAACCTGGCAAAATCGGTTACGGTTGAGTAA-3' and the complementary oligonucleotide into the *Sma*I site of pPY190, creating pGRG60). All mutant attachment sites were variants of that sequence (as described in the text). The challenge phage assay was carried out as described previously [12], using plasmid pCYB1-TnsD or mutant variants of that plasmid to express TnsD, selecting for kanamycin-resistant P22 lysogens. Expression of TnsD proteins was induced with 1 mM IPTG. Results are expressed in lysogens/cell. TnsC was expressed constitutively from pGRG63, a plasmid constructed by cutting pCW15[4] with *Sap*I and *Bs*tXI, blunting and religating the plasmid, to eliminate the TnsAB genes.

## Competing interests

The authors declare that they have no competing interests.

## Authors' contributions

RM, GJM, LY and NLC designed the experiments. RM, GJM, LY and CL performed the experiments. RM, GJM and NLC wrote the manuscript.

## Supplementary Material

Additional file 1 **TnsD protein binds to *attTn7*-like sequences within the *glmS *analog of *Drosophila gfat-1*, and the *gfat-2 *and *glmS *analog of zebrafish *gfpt-1***. Click here for file
