# Supplementary figures and images for: Characterization of the TnsD-attTn7 complex that promotes site-specific insertion of Tn7
Source: Mob DNA. 2010 Jul 23;1:18. doi: 10.1186/1759-8753-1-18 (PMC2918618; doi:10.1186/1759-8753-1-18)

Additional Figure 1

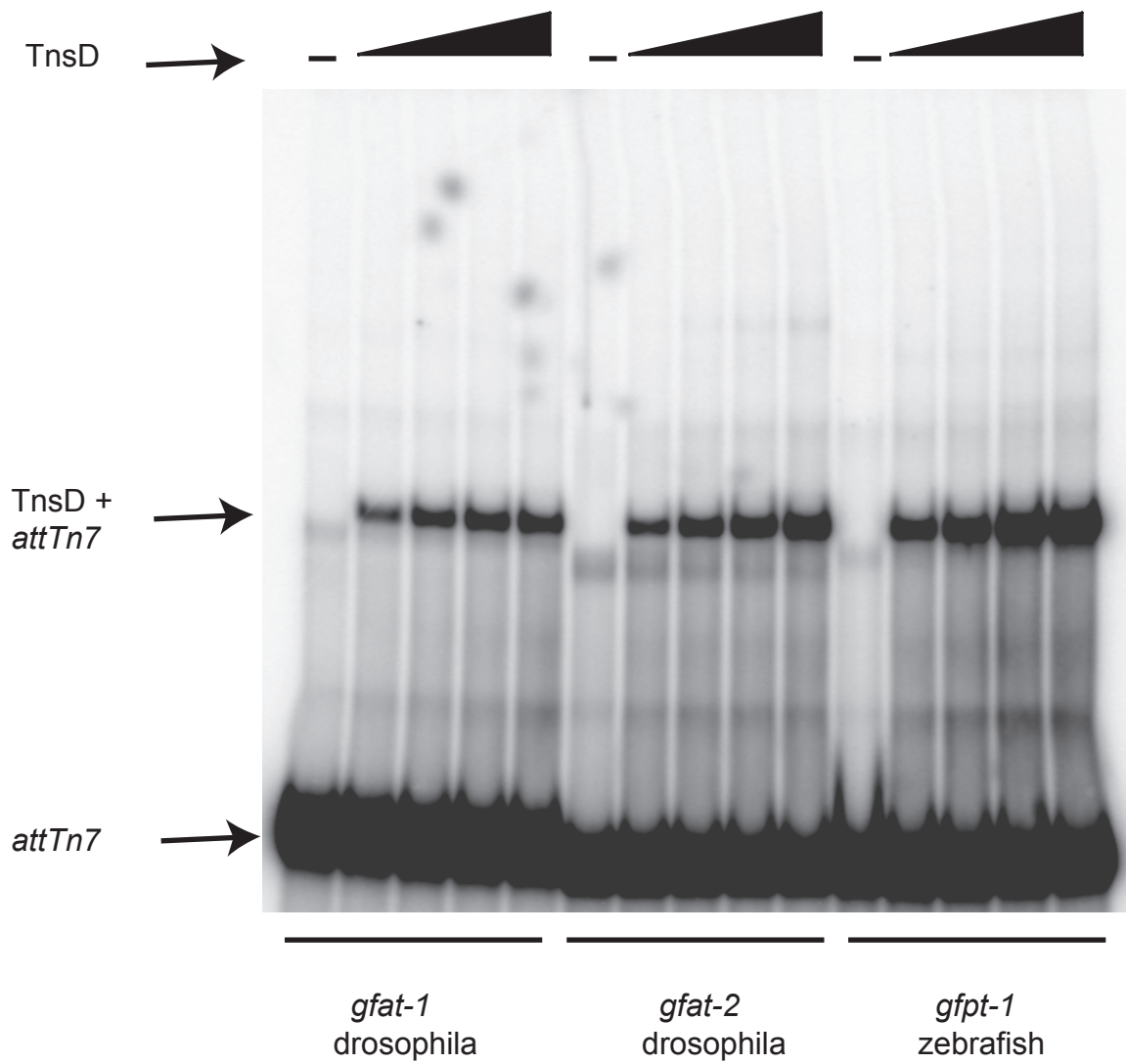

Supplement: Additional file 1 — TnsD protein binds to attTn7-like sequences within the glmS analog of Drosophila gfat-1, and the gfat-2 and glmS analog of zebrafish gfpt-1. [file 1759-8753-1-18-S1.PDF]
